# New Adducts of Iriflophene and Flavonoids Isolated from *Sedum aizoon* L. with Potential Antitumor Activity

**DOI:** 10.3390/molecules22111859

**Published:** 2017-11-02

**Authors:** Mingxiao Li, Zheyuan Qi, Yimeng Hao, Chongning Lv, Lingyun Jia, Jing Wang, Jincai Lu

**Affiliations:** Department of Medicinal Plants, school of Traditional Chinese Materia Medica, Shenyang Pharmaceutical University, Shenyang 110016, China; mingxiaoli0928@126.com (M.L.); sevensc@126.com (Z.Q.); haoyimeng@me.com (Y.H.); lcnmi@outlook.com (C.L.); jialingyun2003@126.com (L.J.); wangjingyk@126.com (J.W.)

**Keywords:** *Sedum aizoon* L., iriflophene, flavonoids, antitumor

## Abstract

Four new special compounds with character of an iriflophene unit and a flavonoid unit connecting via a furan ring were isolated from the roots of *Sedum aizoon* L. Their corresponding structures were elucidated on the basis of spectroscopic analysis. The in vitro anti-proliferative activities against BXPC-3, A549, and MCF-7 tumor cell lines were evaluated. Compounds **3** and **4** exhibited moderate cytotoxic activities with IC_50_ ranging from 24.84 to 37.22 μmol L^−1^, which was capable for further drug exploration.

## 1. Introduction

**Figure 1:**
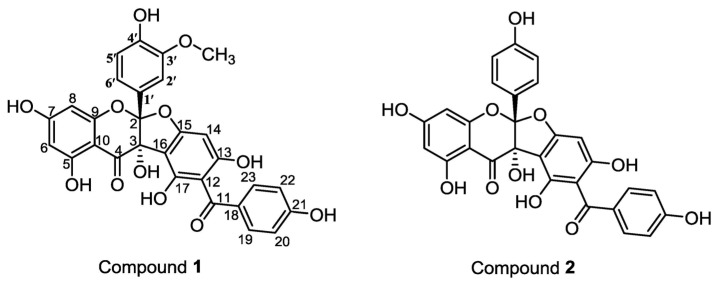

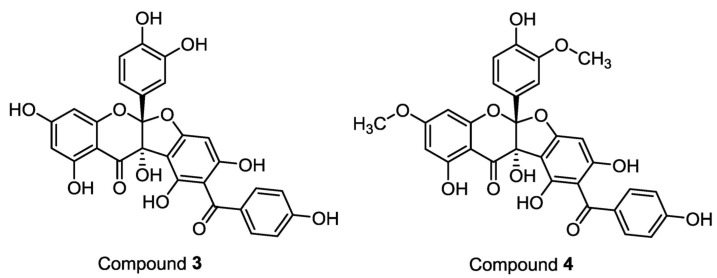
Chemical structures of **1**–**4**.

*Sedum aizoon* L., is an endemic plant, named ‘jingtiansanqi’ in folk medicine. It is distributed in Japan, North Korea, Mongolia, and China. The whole plant is used as a traditional medicine to treat traumatism, hemorrhage, palpitation, and neurasthenia [1,2,3]. Previously, the phytochemical constituents of *Sedum aizoon* L. have been extensively reported but only restricted to the aerial part [4,5,6,7,8,9]. So far, no investigation has been reported regarding the chemical constituents and biological activities of the underground part. In order to find new biologically active compounds, we extracted the roots of *Sedum aizoon* L. and four new special flavonoids were obtained and identified with character of an iriflophene unit and a flavonoid unit connecting via a furan ring (Figure 1). These rare dimers were discovered for the first time. The anti-proliferative activities in vitro against BXPC-3, A549, and MCF-7 tumor cell lines were evaluated by MTT assay.

## 2. Results and Discussion

The ethanol extract of *Sedum aizoon* L. was concentrated and stored at room temperature to yield a crude extract with sediment separated out at the bottom. The sediment was presumed to have low polarity because it dissolved in ethanol but separated out during concentration. Spectroscopic analysis of purified compounds led to the structures of Compounds **1**–**4**. The ^1^H-NMR and ^13^C-NMR, IR, UV, HRESIMS, DEPT, HSQC, HMBC, and CD of Compounds **1**–**4** are available as supplementary materials

Compound **1**, a yellow powder, showed quasi-molecular ions at *m/z* 561.1005 [M + H]^+^ (calcd. for C_29_H_21_O_12_, 561.1028) in HRESIMS spectrum. A broad and intense IR absorption band centered at 3412 cm^−1^ confirmed the presence of hydroxyl groups while an intense band with a shoulder at 1633 cm^−1^ showed the presence of conjugated carbonyl functionalities [10]. The ^1^H NMR spectrum for Compound **1** in dry DMSO-*d*_6_ displayed an isolated proton at *δ*_H_ 3.60 (3H, *s*, OCH_3_) corresponding to the characteristic of methoxyl. Two singlets at *δ*_H_ 5.86 (1H, *s*, H-6) and *δ*_H_ 5.77 (1H, *s*, H-8) indicated a disubstituted ring of a flavonoid while three groups of aromatic at *δ*_H_ 6.61 (1H, *dd*, *J* = 8.3, 1.9 Hz, H-6′), *δ*_H_ 6.66 (1H, *d*, *J* = 8.3 Hz, H-5′), and 6.76 (1H, *d*, *J* = 1.9 Hz, H-2′) assigned to a trisubstituted phenyl moiety. Two pairs of aromatic proton at *δ*_H_ 7.72 (2H, *d*, *J* = 8.7 Hz) and *δ*_H_ 6.85 (2H, *d*, *J* = 8.7 Hz) suggested the presence of E ring in iriflophene unit, while a single proton singlet at *δ*_H_ 6.03 (1H, *s*, H-14) was associated with the single hydrogen on the penta-substituted benzene ring.

The ^13^C NMR data showed 27 resonances, two of which had double intensities indicative of carbons on para-disubstituded aromatic rings. The DEPT (135 spectrum) data confirmed the presence of methane carbons at *δ*_C_ 132.06 (C-19, 23), 119.05 (C-6′), 114.83 (C-20, 22), 114.61 (C-5′), 111.62 (C-2′), 97.66 (C-14), 96.85 (C-6), 95.23 (C-8), and a methoxyl at *δ*_C_ 55.61 (OCH_3_-3′). The presence of seven oxygenated aromatic carbons was inferred from the carbon resonances at *δ*_C_ 168.50 (C-7), 163. 29 (C-5), 162.05 (C-21), 160.69 (C-15), 160.45 (C-9), 159.62 (C-17) and 157.70 (C-13); and the carbon resonances *δ*_C_ 147.63 (C-4′) and *δ*_C_ 146.72 (C-3′) indicated the presences of two oxygenated ortho-carbons [10]. A saturated quaternary carbon with an oxygen atom at *δ*_C_ 79.89 (C-3) was readily characterized, while the dioxygenated carbon at *δ*_C_ 117.01(C-2) was identified by comparison with the chemical shifts of dihydroflavonol moieties from the daphnodorins isolated from *Daphne odora* Thunb [11,12,13].

**Figure 2:**
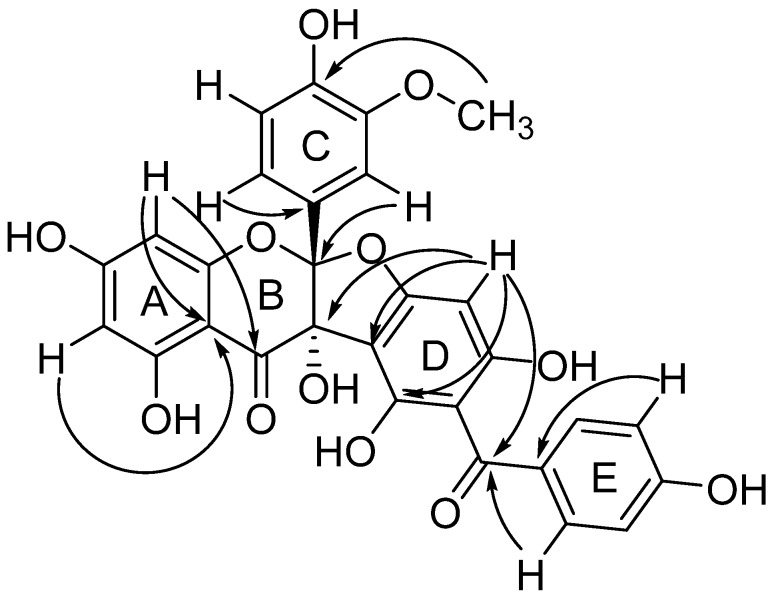
Key HMBC correlations (H→C) observed for Compound **1**.

The linkage between carbons and hydrogen was characterized by the HSQC while the HMQC data effectively positioned the hydroxyl groups and all non-protonated carbons. The hydrogen at *δ*_H_ 5.86 (H-6) showed correlations to the carbons at *δ*_C_ 168.7 (C-7), 163.3 (C-5), 98.1 (C-10), 95.2 (C-8), while the one at *δ*_H_ 5.77 (H-8) exhibited correlations to the carbons at *δ*_C_ 191.3 (C-4), 168.5 (C-7), 160.5 (C-9), 98.1 (C-10), and 96.9 (C-6) which confirmed the structure of ring A as a common disubstituted ring of a flavonoid. The structure of ring C was confirmed as a trisubstituted phenyl connected to the C-2 by a series correlations of the hydrogen at *δ*_H_ 6.61 (H-6′) to the carbons at *δ*_C_ 147.6 (C-4′), 117.0 (C-2), 111.6 (C-2′); the hydrogen at *δ*_H_ 6.66 (H-5′) to the carbons at *δ*_C_ 146.7 (C-4′), 124.8 (C-1′) and the one at *δ*_H_ 6.76 (H-2′) to the carbons at *δ*_C_ 147.6 (C-4′), 124.8 (C-1′), 119.1 (C-6′), and 117.0 (C-2). The single hydrogen at *δ*_H_ 6.03 (H-14) showed correlations to the carbons at *δ*_C_ 191.3 (C-11), 106.3 (C-16), 103.2 (C-12), and 79.9 (C-3) which showed the penta-substituted benzene ring D was connected to the feature structure C-2 and C-3. The hydrogen at *δ*_H_ 7.72 (H-19, 23) displayed correlations to the carbons at *δ*_C_ 191.3 (C-11) and 162.1 (C-21); and *δ*_H_ 6.85 (H-20, 22) exhibited correlations to the carbons at *δ*_C_ 162.1 (C-21) and 129.7 (C-18) which corroborated the linkage of ring E as a disubstituted ring connected to the ring D via a carbonyl [14,15,16,17]. The *δ*_H_ 6.03 (H-14) showed no correlation to the carbons at *δ*_C_ 159.62 which excluded the possibility that C-11 connected to C-14 (Figure 2). 

Thus, the structure of Compound **1** was assigned as 1,3,8,10,10b-pentahydroxy-5a-(4-hydroxy-3-methoxyphenyl)-9-(4-hydroxybenzoyl)-5a,10b-dihydro-11*H*-benzofuro[2,3-b]chromen-11-one, an iriflophene unit and an isorhamnetin unit connecting via a furan ring [18,19].

Compound **2** was obtained as a yellowish amorphous solid. The molecular formula was determined to be C_28_H_18_O_11_ from HRESIMS which showed a quasi-molecular ion peak at *m/z*: 531.0907 [M + H]^+^ (calcd. for C_28_H_19_O_11_, 531.0922). Its IR, ^1^H NMR, and ^13^C NMR spectrum was alike with Compound **1**, revealed a similar structure. However, the observation of four pairs of aromatic proton of ^1^H at *δ*_H_ 7.70 (2H, *d*, *J* = 8.4 Hz, H-19, 23), 7.06 (2H, *d*, *J* = 8.4 Hz, H-2′ 6′), 6.85 (2H, *d*, *J* = 8.4 Hz, H-20, 22), 6.67 (2H, *d*, *J* = 8.4 Hz, H-3′ 5′) as well as ^13^C at *δ*_C_ 132.54 (C-19, 23), 114.93 (C-3′ 5′), 128.57 (C-2′ 6′), 115.36 (C-20, 22) suggested that there were two disubstituted rings instead of a trisubstituted phenyl moiety as such structures would show carbon proton between *δ*_C_ 144-148. According to the analysis of HSQC and HMBC, the structure of Compound **2** was elucidated as 1,3,8,10,10b-pentahydroxy-9-(4-hydroxybenzoyl)-5a-(4-hydroxyphenyl)-5a,10b-dihydro-11*H*-benzofuro[2,3-b]chromen-11-one, an iriflophene unit and a kaempferol unit connecting via a furan ring [20].

Compound **3** was isolated as a yellowish amorphous solid. The HRESIMS showed a quasi-molecular ion peak at *m/z*: 547.0858 [M + H]^+^ (calcd. for C_28_H_19_O_12_, 547.0871), corresponding to a molecular formula of C_28_H_18_O_12_. Its IR, ^1^H NMR, and ^13^C NMR spectrum was similar to Compound **1** but without the signal of methoxyl, which suggested that Compound **3** has the same frameworks as **1** and the methoxy should turn into a hydroxyl. The HSQC and HMBC data supported the postulate. From the above information, the structure of Compound **3** was assigned as 5a-(3,4-dihydroxyphenyl)-1,3,8,10,10b-pentahydroxy-9-(4-hydroxybenzoyl)-5a,10b-dihydro-11*H*-benzofuro[2,3-b]chromen-11-one, an iriflophene unit, and a quercetin unit connecting via a furan ring [21].

Compound **4** was obtained as a yellowish amorphous solid. The molecular formula was determined to be C_30_H_22_O_12_ from HRESIMS which showed a quasi-molecular ion peak at *m/z*: 575.1162 [M + H]^+^ (calcd. for C_30_H_23_O_12_, 575.1184). Its IR, ^1^H NMR, and ^13^C NMR spectrum was similar to Compound **1**. Another methoxyl at *δ*_H_ 3.77 suggested that a hydroxyl should be replaced by methoxyl. The HMBC correlation from 7-OCH_3_ (*δ*_H_ 3.77) to C-7 (*δ*_C_ 167.88) located the methoxy group at C-7. Therefore, the structure of Compound **4** was elucidated as 1,8,10,10b-tetrahydroxy-5a-(4-hydroxy-3-methoxyphenyl)-9-(4-hydroxybenzoyl)-3-methoxy-5a,10b-dihydro-11*H*-benzofuro[2,3-b]chromen-11-one, an iriflophene unit and a rhamnazin unit connecting via a furan ring [22].

**Figure 3:**
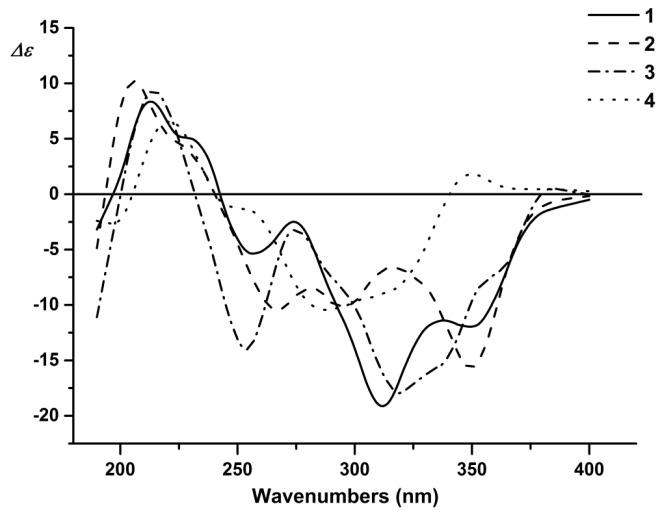
The CD spectra of Compounds **1**–**4**.

The determination of the absolute configuration of C-2 and C-3 in Compounds **1**–**4** was established by circular dichroic (CD) spectra (Figure 3). The CD spectra showed a negative cotton effect similar to that of daphnodorin F and H at 275 and 321 nm. Therefore, the absolute configuration of C-2 and C-3 was assigned as 2*S*, 3*R* [23,24]. 

Subsequently, the isolated Compounds **1**–**4** were evaluated for in vitro cytotoxicity against BXCP-3, MCF-7, and A549 tumor cell lines. The result revealed that Compounds **3** and **4** exhibited moderate cytotoxic activities to all three cell lines with IC_50_ ranging from 24.84 to 37.22 μmol L^−1^, as shown in Table 1. With those distinct frameworks and promising activities, Compounds **3** and **4** can be considered as potential lead compounds for the further structural modification and biological evaluation.

## 3. Materials and Methods

### 3.1. General Procedures

Optical rotations were determined on an Anton Paar MCP-200 polarimeter (Anton Paar, Graz, Austria) in MeOH at 20 °C. UV spectra were obtained on a UV-1700 visible apectrophotometer (Shimadzu, Kyōto, Japan). IR spectra were recorded using a Bruker IFS-55 IR spectrometer with KBr disks. NMR experiments were performed on a Bruker 400 MHz 600 MHz AV Ш HD spectrometers (Bruker Biospin, Rheinstetten, Germany). HR-ESI-MS carried out on an Agilent Technologies 6540 UHD accurate mass Q-TOF MS apparatus (Agilent, Santa Clara, CA, USA). ECD spectra were recorded on a BioLogic ECD spectrometer (BioLogic, Pariset, France). Column chromatography (CC) was performed with silica gel (100–200 mesh, 200–300 mesh, Qingdao Marine Chemical, Inc., Qingdao, China). Silica GF_254_ (10–40 μm; Qingdao Marine Chemical, Inc., Qingdao, China) and Silica G (10–40 μm; Qingdao Marine Chemical, Inc., Qingdao, China) were used for TLC. Spots were observed by UV light or by spraying with 10% H_2_SO_4_-EtOH followed by heating. Preparative HPLC was performed on a Shimadzu 20A system with a YMC-pack (ODS-A, 20 × 250 mm, 5 μm) running with a flow rate of 3.5 mL min^−1^.

### 3.2. Plant Material

The roots of *S. aizoon* L. were collected at Heze, Shandong Province, China, in August 2016 and identified by Prof. Jincai Lu (School of Traditional Chinese Materia Medica, Shenyang Pharmaceutical University, China). A voucher specimen (No.20160930) was deposited in the Herbarium of Shenyang Pharmaceutical University.

### 3.3. Extraction and Isolation

The dry roots of *S. aizoon* (17 kg) was extracted with 70% ethanol (136 L × 3 times) and filtered. The filtrate was concentrated under reduced pressure and stored at room temperature to yield a crude extract (20 L) with sediment separated out at the bottom. The crude extract was then centrifuged to separate sediment. The sediment (220 g) was chromatographed on a 2000 g silica gel column, eluting with CH_2_Cl_2_/CH_3_OH (1:0, 50:1, 35:1, 20:1, 10:1, 1:1) to obtain six fractions (fraction 1–6). The fraction 2 (6.0 g) of CH_2_Cl_2_/CH_3_OH (50:1) continued silica gel column elution with petroleum ether-EtOAc (4:1, 2:1, 1:1). The fraction 2-2(1.0 g) of petroleum ether-EtOAc (2:1) was further purified by preparative HPLC (YMC-pack ODS-A, 20 × 250 mm, 5 μm, 60% MeOH in H_2_O) to afford Compound **1** (8 mg). The fraction 4 (5.0 g) of CH_2_Cl_2_/CH_3_OH (20:1) was chromatographed on silica gel column, eluting with CH_2_Cl_2_/CH_3_OH (30:1, 10:1, 1:1). Fraction 4–2 (1.0 g) continued silica gel column elution with CH_2_Cl_2_/CH_3_OH (15:1) and further purified by preparative HPLC (YMC-pack ODS-A, 20 × 250 mm, 5 μm, 60% MeOH in H_2_O) to afford Compound **2** (11 mg). Fraction 4–3 (900 mg) was applied to preparative HPLC (YMC-pack ODS-A, 20 × 250 mm, 5 μm, 60% MeOH in H_2_O) to obtain Compound **3** (20 mg).The fraction 6 was subjected to silica gel column, eluting with CH_2_Cl_2_/CH_3_OH (30:1, 10:1, 1:1), and fraction 6–2 (800 mg) continued silica gel column elution with CH_2_Cl_2_/CH_3_OH (25:1) to yield Compound **4** (13 mg).

### 3.4. Compound Characterization

Compound **1**: Yellowish amorphous solid (MeOH); ${\left[\alpha \right]}_{D}^{20}$-13.06 (*c* 0.1033, MeOH); UV *λ*_max_(MeOH): 300(3.98) nm, 342(3.80) nm; IR(KBr) *ν*_max_/cm^−1^ 3412, 1633, 1515, 1428, 1284, 1170, 1048, 1023. ^1^H and ^13^C NMR data see Table 2; HRESIMS *m/z*: 561.1005 [M + H]^+^ (calcd. for C_29_H_21_O_12_, 561.1028).

Compound **2**: Yellowish amorphous solid (MeOH); ${\left[\alpha \right]}_{D}^{20}$-11.58 (*c* 0.0944, MeOH); UV *λ*_max_(MeOH): 301(3.55) nm, 345(3.57) nm; IR(KBr) *ν*_max_/cm^−1^ 1632, 1512, 1439, 1274, 1170, 1044, 1023. ^1^H and ^13^C NMR data see Table 2; HRESIMS *m/z*: 531.0907 [M + H]^+^ (calcd. for C_28_H_19_O_11_, 531.0922).

Compound **3**: Yellowish amorphous solid (MeOH); ${\left[\alpha \right]}_{D}^{20}$-18.39 (*c* 0.1142, MeOH); UV *λ*_max_(MeOH): 300(4.28) nm, 343(4.26) nm; IR(KBr) *ν*_max_/cm^−1^ 3396, 1632, 1510, 1403, 1272, 1169, 1022. ^1^H and ^13^C NMR data see Table 3; HRESIMS *m/z*: 547.0858 [M + H]^+^ (calcd. for C_28_H_19_O_12_, 547.0871).

Compound **4**: Yellowish amorphous solid (MeOH); ${\left[\alpha \right]}_{D}^{20}$-10.69 (*c* 0.0842, MeOH); UV *λ*_max_(MeOH): 301(3.10) nm, 340(2.98) nm; IR(KBr) *ν*_max_/cm^−1^ 3405, 1632, 1514, 1282, 1129, 1047, 1023. ^1^H and ^13^C NMR data see Table 3; HRESIMS *m/z*: 575.1162 [M + H]^+^ (calcd. for C_30_H_23_O_12_, 575.1184).

### 3.5. Cytotoxicity Assay

The cytotoxicity assay of Compounds **1**–**4** was performed via the MTT method using three kinds of human cancer cell lines, including BXPC-3, MCF-7, and A549 (American Type Culture Collection, Rockville, MD, USA). BXPC-3 and MCF-3 were grown in Dulbecco’s modified Eagle medium (DMEM) while A549 was grown in 1640 medium, supplemented with 10% fetal bovine serum and cultured at a density of 6 × 10^4^ cells mL^−1^ in 96-well microtiter plate for overnight. Compounds were dissolved in DMSO at five different concentrations and subsequently added to the wells in triplicates. After incubation at 37 °C with 5% CO_2_ for 72 h, the cells were incubated with 15 μL of MTT (5 mg mL^−1^) for another 4 h. The residual liquid was removed while 150 μL DMSO was added. The absorbance was detected using a microplate reader at 492 nm. All tests and analyses were carried out in three independent assays with DMSO (final concentration of 0.1%) and 5-FU applied as the blank control and positive control, respectively. The anti-proliferative activities were expressed as the IC_50_ value (50% inhibitory concentration).

## 4. Conclusions

In the present study, four new special adducts of iriflophene and flavonoids connecting via a furan ring were discovered. The dimers of iriflophene and flavonoids were reported for the first time, which also enriched the chemical constituents of the Crassulaceae family. Previously, only a few bioflavonoids analogues were found in *Daphane odora* but their biological activities were indistinctive [13]. In our research, Compounds **3** and **4** exhibited moderate cytotoxic activities against BXPC-3, A549, and MCF-7 tumor cell lines. Their activities were better than the uncombined unit maybe due to the furan ring connections. Therefore, the special flavonoids isolated from *Sedum aizoon* L. were meaningful as potential antitumor leading compounds in the medicine industry.

## Figures and Tables

**Table 1:** Cytotoxicity activities of Compounds **1**–**4** from *Sedum aizoon* L.

Compound	IC_50_ (μmol L^−1^)		
	BXPC-3	MCF-7	A549
**1**	>100	>100	>100
**2**	>100	>100	>100
**3**	24.84	35.89	37.20
**4**	31.22	33.90	26.11
**5-FU**	15.81	17.36	2.96

5-FU: positive control.

**Table 2:** ^1^H NMR and ^13^C NMR data of Compounds **1** and **2** in DMSO-*d*_6_.

Position	1 ^a^			2 ^b^
	*δ*_C_	*δ*_H_	*δ*_C_ Type COSY	*δ*_H_
4	191.25		191.64	
11	191.02		191.64	
7	168.50		168.26	
5	163.29		163.75	
21	162.05		162.57	
15	160.69		161.15	
9	160.45		160.75	
17	159.62		160.07	
13	157.7		158.10	
4′	147.63		158.77	
3′	146.72		114.93	
19, 23	132.06	7.72 (*d*, *J* = 8.7 Hz, 2H)	132.54	7.70 (*d*, *J* = 8.4 Hz, 2H)
18	129.70		130.00	
1′	124.81		124.67	
6′	119.05	6.61 (*dd*, *J* = 8.3, 1.9 Hz,1H)	128.57	7.06 (*d*, *J* = 8.4 Hz, 2H)
2	117.01		117.76	
20, 22	114.83	6.85 (*d*, *J* = 8.6 Hz, 2H)	115.36	6.85 (*d*, *J* = 8.4 Hz, 2H)
5′	114.61	6.66 (*d*, *J* = 8.3 Hz, 1H)	114.93	6.67 (*d*, *J* = 8.4 Hz, 2H)
2′	111.62	6.76 (*d*, *J* = 1.9 Hz, 1H)	128.57	
16	106.31		106.84	
12	103.23		104.00	
10	98.08		98.76	
14	97.66	6.03 (*s*, 1H)	97.96	6.04 (*s*, 1H)
6	96.85	5.86 (*s*, 1H)	97.10	5.90 (*d*, *J* = 2.0 Hz, 1H)
8	95.23	5.77 (*s*, 1H)	95.28	5.80 (*d*, *J* = 2.0 Hz, 1H)
3	79.89		80.32	
3′-OCH_3_	55.61	3.60 (*s*, 3H)		

^a 13^C 150 Hz, ^b 13^C 100 Hz.

**Table 3:** ^1^H NMR and ^13^C NMR data of Compounds **3** and **4** in DMSO-*d*_6_.

Position	3 ^a^			4 ^b^
	*δ*_C_	*δ*_H_	*δ*_C_ Type COSY	*δ*_H_
4	192.53		192.89	
11	191.55		191.64	
7	167.30		167.88	
5	163.66		163.25	
21	162.64		162.52	
15	161.36		161.32	
9	160.46		161.10	
17	160.02		160.29	
13	157.64		157.50	
3′	146.91		148.20	
4′	144.87		147.26	
19, 23	132.58	7.71 (*d*, *J* =8.7 Hz, 2H)	132.56	7.73 (*d*, *J* = 8.6 Hz, 2H)
18	129.92		130.18	
1′	124.97		124.85	
6′	118.40	6.53 (*dd*, *J* = 8.3, 2.2 Hz,1H)	119.36	6.60 (*dd*, *J* = 8.6, 2.0 Hz, 1H)
2	117.91		117.94	
20, 22	115.43	6.87 (*d*, *J* = 8.7 Hz,2H)	115.33	6.86 (*d*, *J* = 8.6 Hz, 2H)
5′	115.13	6.63 (*d*, *J* = 8.3 Hz, 1H)	115.15	6.67 (*d*, *J* = 8.3 Hz, 1H)
2′	114.80	6.70 (*d*, *J* = 8.3 Hz, 1H)	111.91	6.75 (*d*, *J* = 2.0 Hz, 1H)
16	106.94		106.80	
12	104.26		103.79	
10	98.98		99.92	
14	97.87	6.06 (*s*, 1H)	98.17	6.04 (*s*, 1H)
6	96.79	5.95 (*d*, *J* = 2.0 Hz, 1H)	95.81	6.13 (*d*, *J* = 1.7 Hz, 1H)
8	94.97	5.85 (*d*, *J* = 2.0 Hz, 1H)	93.75	6.07 (*d*, *J* = 1.7 Hz, 1H)
7-OCH_3_	94.97		56.07	3.77 (*s*, 3H)
3-OCH_3_	94.97		55.37	3.61 (*s*, 3H)

^a 13^C 100 Hz, ^b 13^C 150 Hz.

## Supplementary Materials

The ^1^H-NMR and ^13^C-NMR, IR, UV, HRESIMS, DEPT, HSQC, HMBC, and CD of Compounds **1**–**4** are available as supplementary materials. Supplementary materials are available online.
