# Supplementary material for: New Adducts of Iriflophene and Flavonoids Isolated from Sedum aizoon L. with Potential Antitumor Activity
Source: Molecules. 2017 Nov 2;22(11):1859. doi: 10.3390/molecules22111859 (PMC6150161; doi:10.3390/molecules22111859)
Supplement: Supplementary file 1 [file molecules-22-01859-s001.pdf]

Supporting information

New adducts of iriflophene and flavonoids isolated from *Sedum aizoon* L. with potential antitumor activity

Mingxiao Li, Zheyuan Qi, Yimeng Hao, Chongning Lv, Lingyun Jia, Jing Wang, Jincai Lu*

Department of Medicinal Plants, school of Traditional Chinese Materia Medica, Shenyang Pharmaceutical University, Shenyang 110016, P.R. China. Email: jincailu@126.com; Fax:+86 024 23986500; Tel: +86 024 23986500

Table of Contents

Figure S1. ^{1}H NMR spectrum (600 MHz, DMSO-d_6) of compound **1**

Figure S2. ^{13}C NMR spectrum (150 MHz, DMSO-d_6) of compound **1**

Figure S3. HMBC spectrum (600 MHz, DMSO-d_6) of compound **1**

Figure S4. HSQC spectrum (600 MHz, DMSO-d_6) of compound **1**

Figure S5. DEPT spectrum (135 spectrum)) of compound **1**

Figure S6. HRESIMS spectrum of compound **1**

Figure S7. IR spectrum of compound **1**

Figure S8. UV spectrum of compound **1**

Figure S9. ^{1}H NMR spectrum (600 MHz, DMSO-d_6) of compound **2**

Figure S10. ^{13}C NMR spectrum (100 MHz, DMSO-d_6) of compound **2**

Figure S11. HMBC spectrum (600 MHz, DMSO-d_6) of compound **2**

Figure S12. HSQC spectrum (600 MHz, DMSO-d_6) of compound **2**

Figure S13. HRESIMS spectrum of compound **2**

Figure S14. IR spectrum of compound **2**

Figure S15. UV spectrum of compound **2**

Figure S16. ^{1}H NMR spectrum (600 MHz, DMSO-d_6) of compound **3**

Figure S17. ^{13}C NMR spectrum (100 MHz, DMSO-d_6) of compound **3**

Figure S18. HMBC spectrum (600 MHz, DMSO-d_6) of compound **3**

Figure S19. HSQC spectrum (600 MHz, DMSO-d_6) of compound **3**

Figure S20. HRESIMS spectrum of compound **3**

Figure S21. IR spectrum of compound **3**

Figure S22. UV spectrum of compound **3**

Figure S23. ^{1}H NMR spectrum (600 MHz, DMSO-d_6) of compound **4**

Figure S24. ^{13}C NMR spectrum (150 MHz, DMSO-d_6) of compound **4**

Figure S25. HMBC spectrum (600 MHz, DMSO-d_6) of compound **4**

Figure S26. HSQC spectrum (600 MHz, DMSO-d_6) of compound **4**

Figure S27. HRESIMS spectrum of compound **4**

Figure S28. IR spectrum of compound **4**

Figure S29. UV spectrum of compound **4**

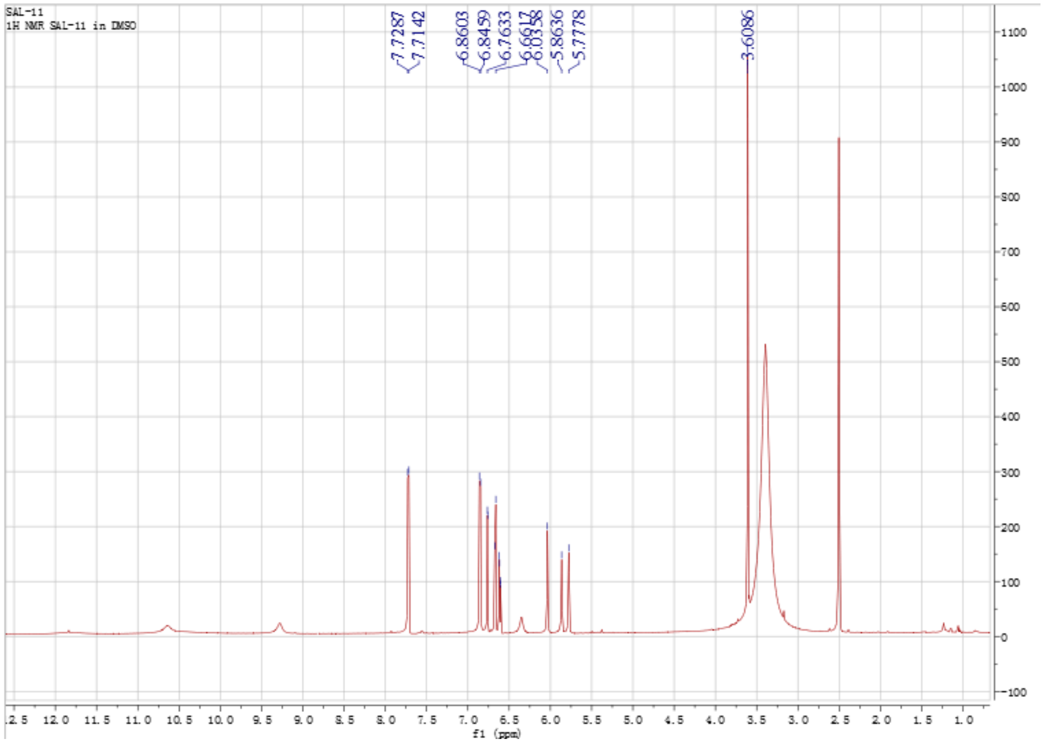

Figure S1. ^{1}H NMR spectrum (600 MHz, DMSO-d_6) of compound **1**

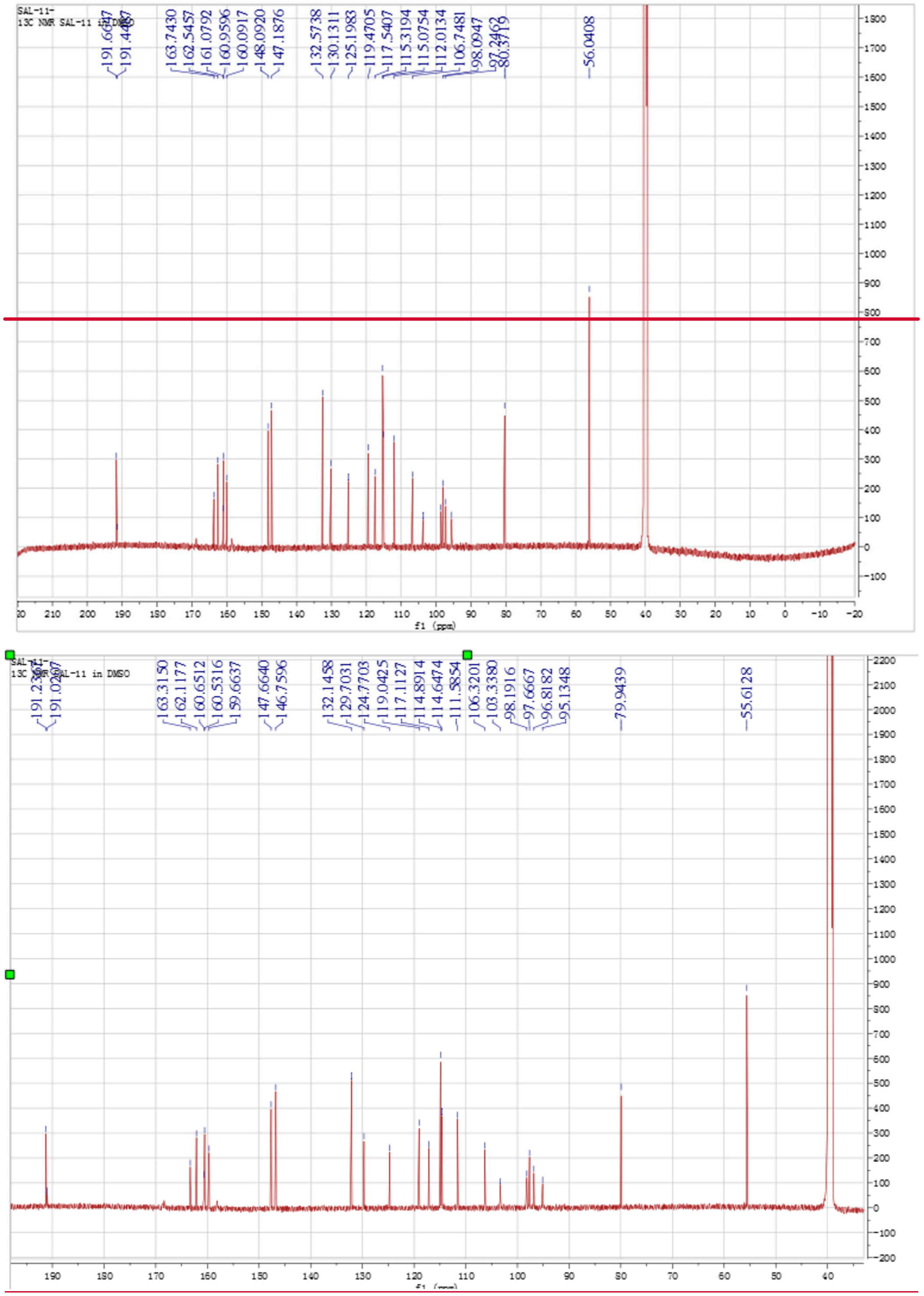

Figure S2. ^{13}C NMR spectrum (150 MHz, DMSO-d_6) of compound **1**

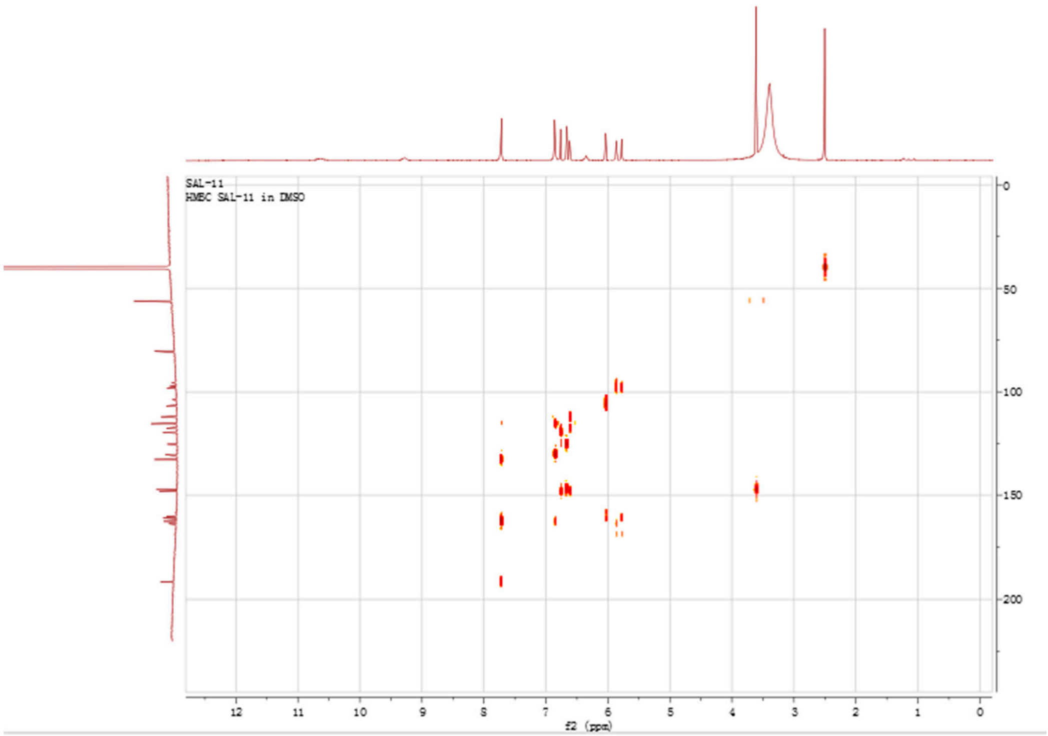

Figure S3. HMBC spectrum (600 MHz, DMSO-d_6) of compound **1**

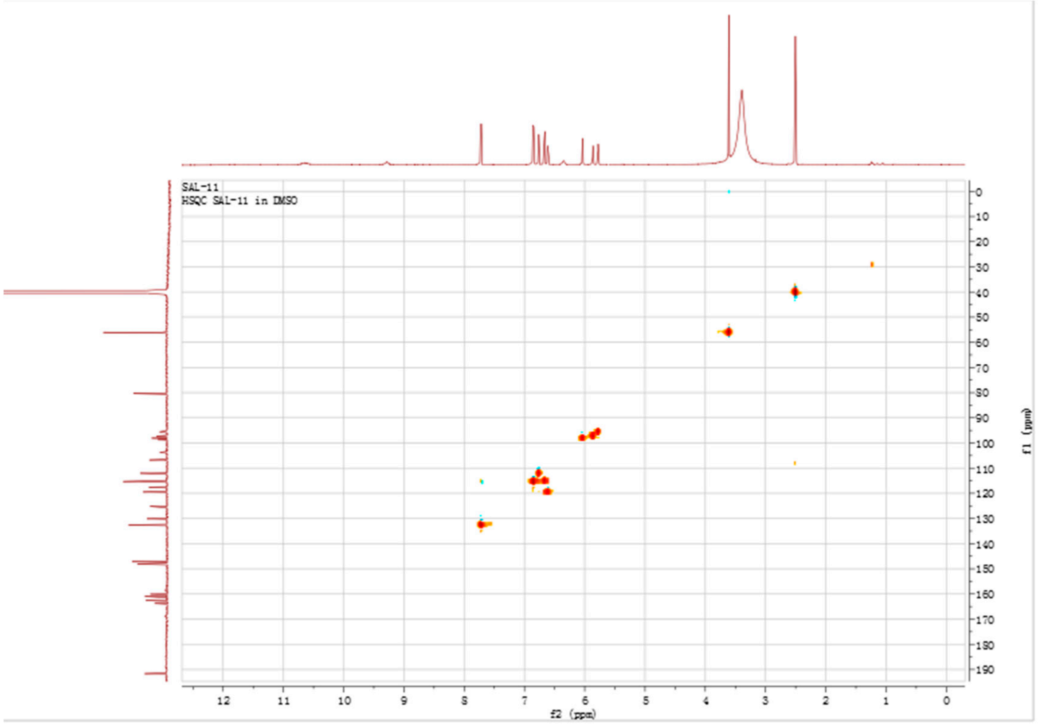

Figure S4. HSQC spectrum (600 MHz, DMSO-d_6) of compound **1**

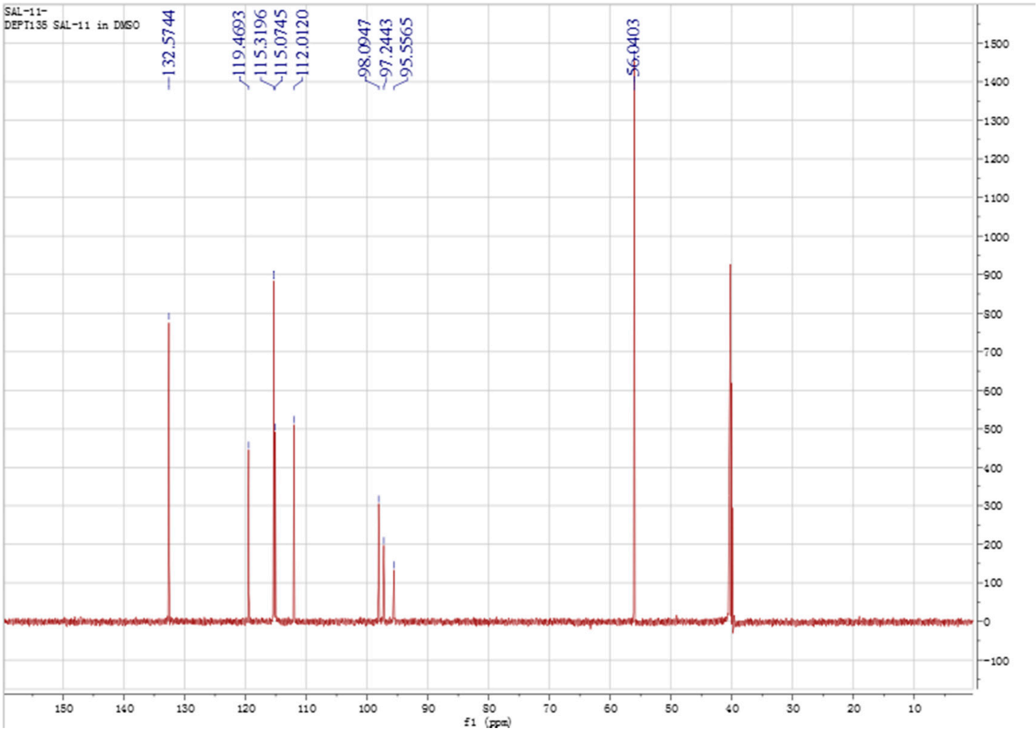

Figure S5. DEPT spectrum (135 spectrum)) of compound **1**

User Spectra

Fragmentor Voltage	Collision Energy	Ionization Mode
75	0	ESI

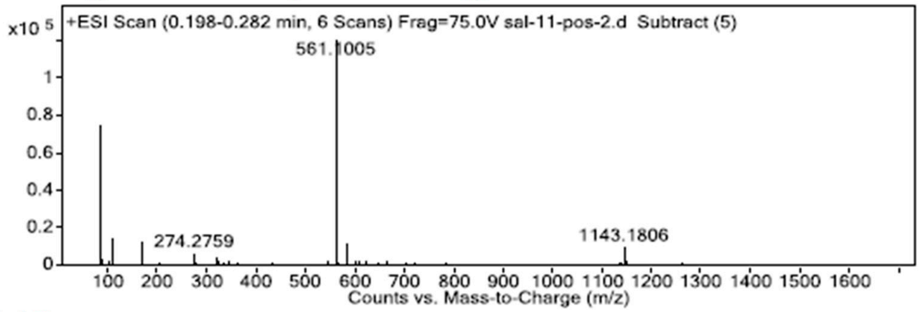

Peak List

m/z	z	Abund	Formula	Ion
85.0596		74599.4		
85.0729		33017.4		
107.0413		14535		
107.056		7475.1		
169.1119		12608		
274.2759		6209		
561.1005	1	120743.1	C29 H21 O12	(M+H)+
561.1328	1	64806		
562.1035	1	36291.6	C29 H21 O12	(M+H)+
562.1367	1	21170.8		
563.1045	1	8555.7	C29 H21 O12	(M+H)+
583.0847		11954.5		
1143.1806	1	9423.1		
1144.1864	1	6203		

Formula Calculator Element Limits

Element	Min	Max
C	0	60
H	0	120
O	0	20
N	0	0
S	0	0
Cl	0	0
P	0	0
B	0	0
F	0	0
Cu	0	0
Co	0	0

Formula Calculator Results

Formula	Best	Mass	Tgt Mass	Diff (ppm)	Ion Species	Score
C29 H20 O12	TRUE	560.0932	560.0955	4.07	C29 H21 O12	90.27
C36 H16 O7		560.0932	560.0896	-6.41	C36 H17 O7	71.69

--- End Of Report ---

Figure S6. HRESIMS spectrum of compound 1

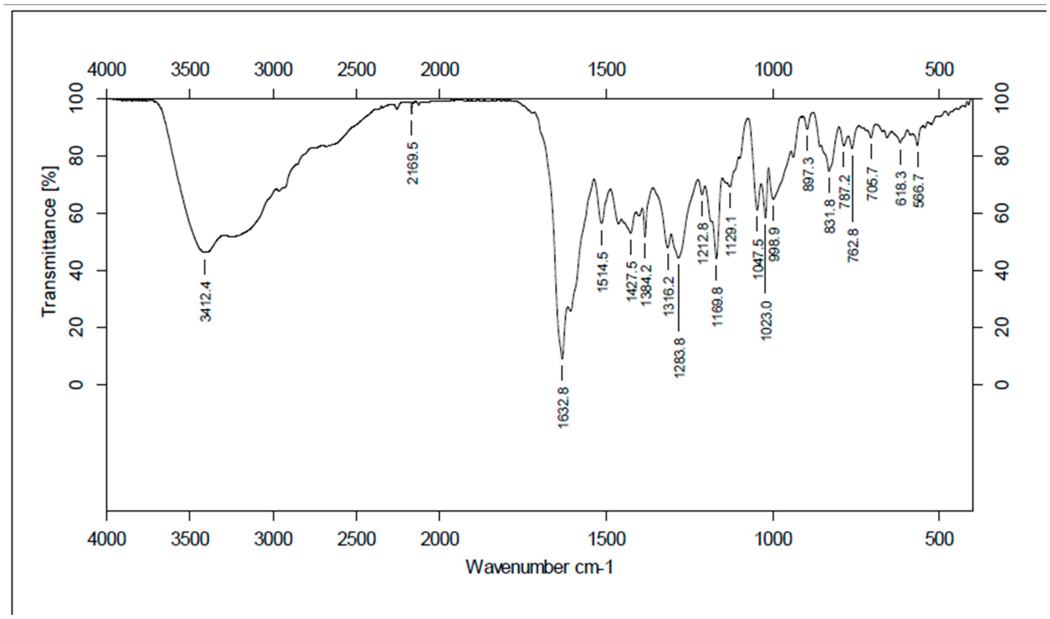

Figure S7. IR spectrum of compound 1

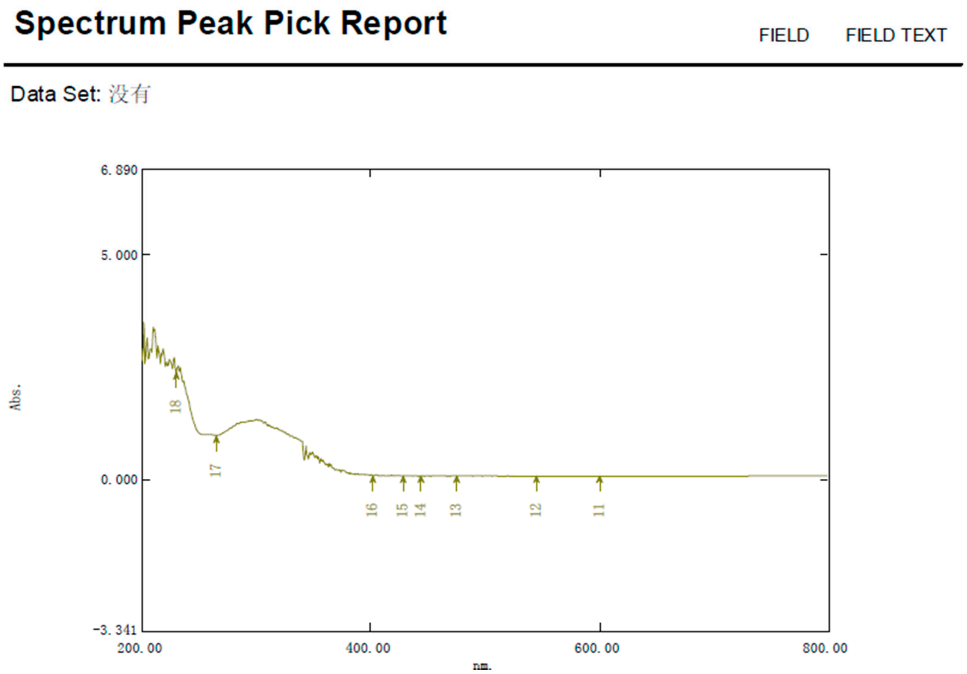

Figure S8. UV spectrum of compound 1

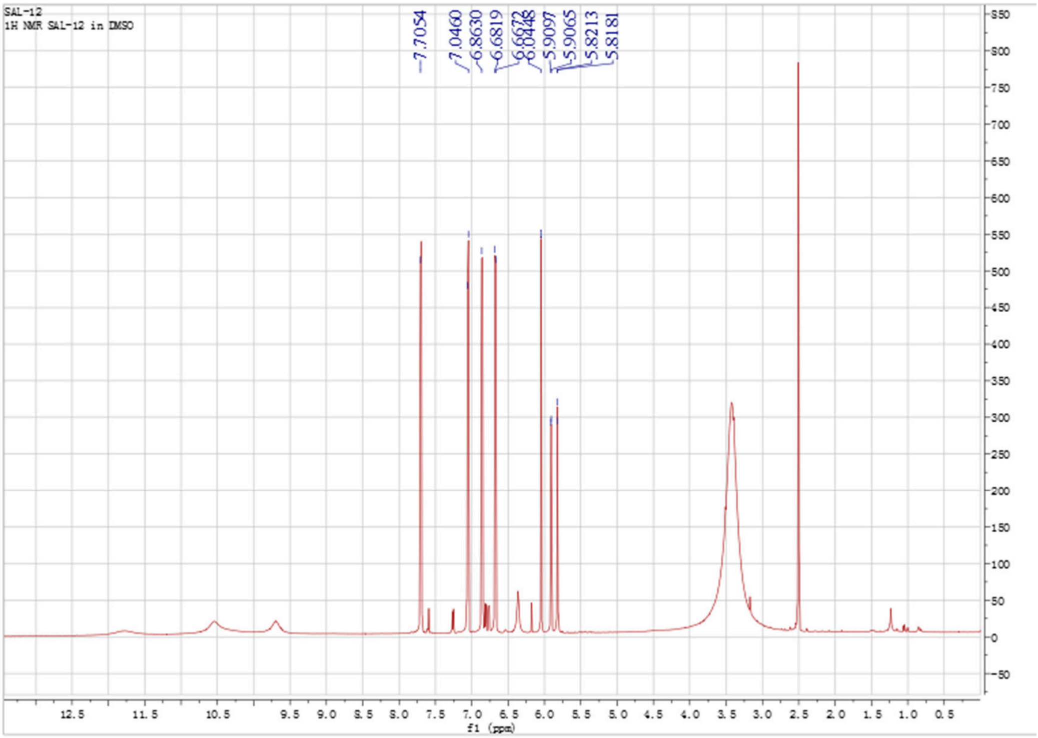

Figure S9. ^{1}H NMR spectrum (600 MHz, DMSO-d_6) of compound **2**

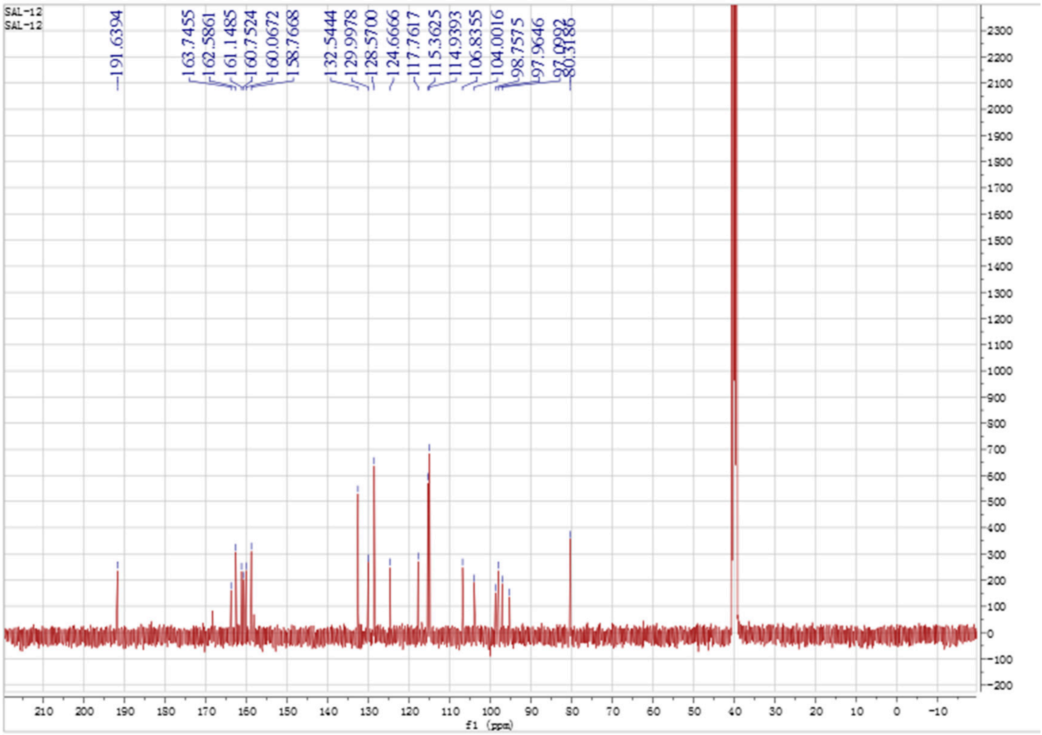

Figure S10. ^{13}C NMR spectrum (100 MHz, DMSO-d_6) of compound **2**

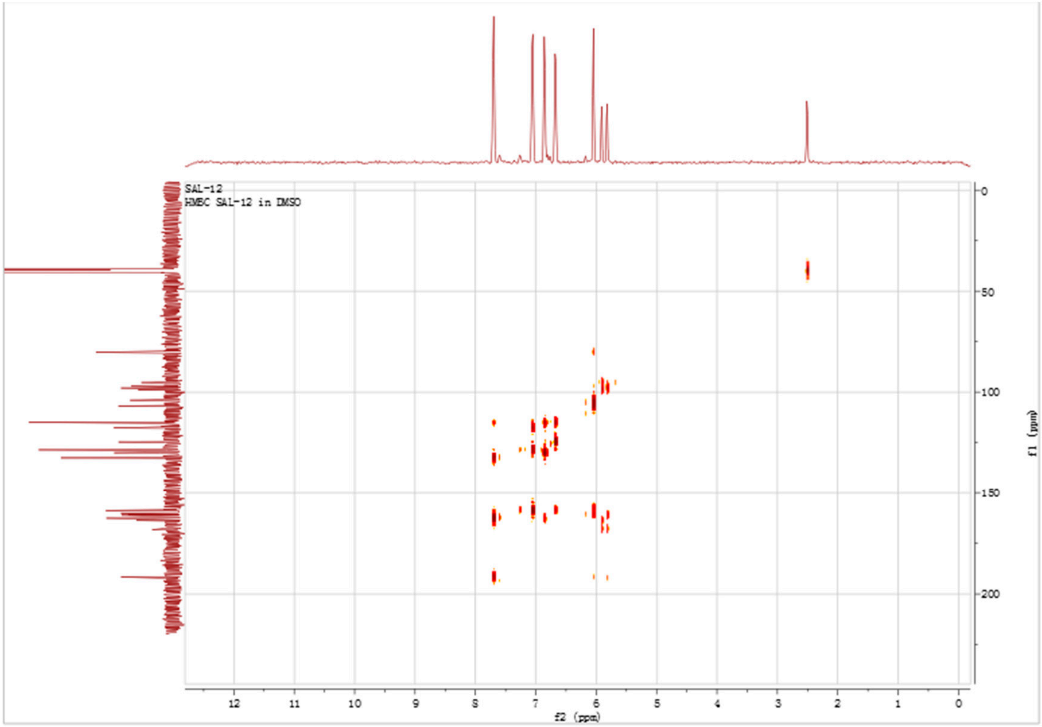

Figure S11. HMBC spectrum (600 MHz, DMSO-d_6) of compound **2**

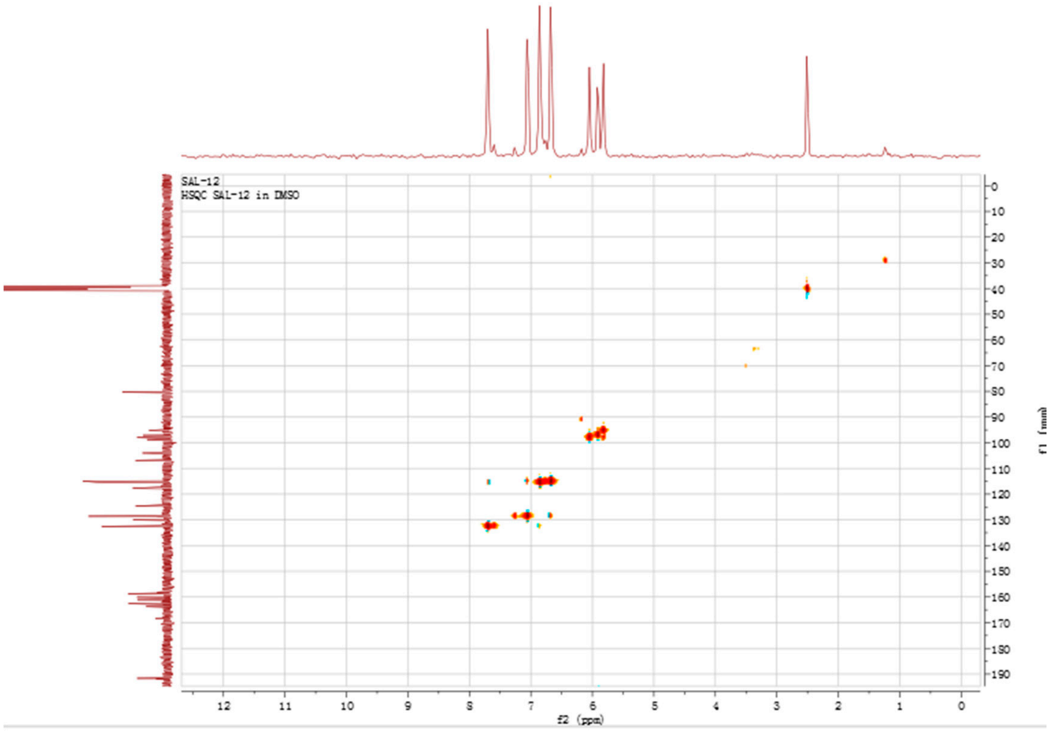

Figure S12. HSQC spectrum (600 MHz, DMSO-d_6) of compound **2**

User Spectra

Fragmentor Voltage	Collision Energy	Ionization Mode
75	0	ESI

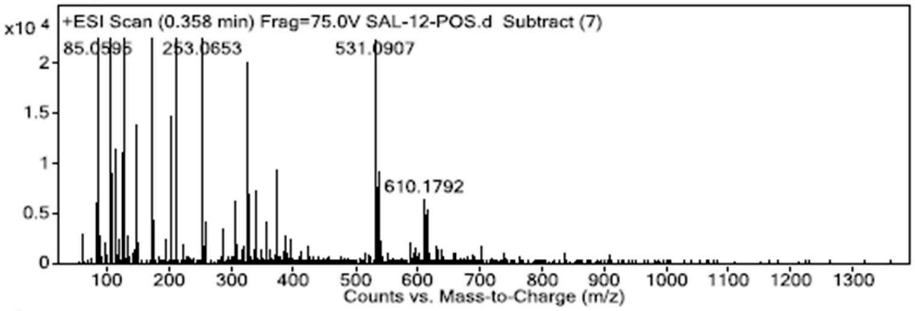

Peak List

m/z	z	Abund	Formula	Ion
82.5202		6139.2		
85.0595		114979.6		
85.0725		53226.6		
103.0337	1	69789.6		
103.049		29159.7		
104.0317	1	12281.6		
107.0416		8984.4		
111.5414		11406.2		
123.5471		11094.2		
124.5467		30290.5		
124.5624		16359.2		
146.0592		13873.3		
146.0778		7952.9		
169.0133		38703		
169.0315		16424		
169.1118		23267.3		
169.1298		9605.8		
201.0334		14803		
210.0399		27838.9		
210.0614		8901.1		
253.0653		36768.4		
253.0885		18095.5		
305.5403		6332.9		
325.1178		20173.9		
325.1421		11270.9		

326.0511		6911.4		
337.1165		7401.2		
338.3423		6002.4		
371.2121		9537.2		
531.0907	1	22430.7	C28 H19 O11	(M+H)+
531.1221		12370.6		
532.0935	1	7615.7	C28 H19 O11	(M+H)+
536.1639		9305.5		
610.1792		6512.5		

Formula Calculator Element Limits

Element	Min	Max
C	0	60
H	0	120
O	0	15
N	0	0
S	0	0
Cl	0	0
P	0	0
B	0	0
F	0	0
Cu	0	0
Co	0	0

Formula Calculator Results

Formula	Best	Mass	Tgt Mass	Diff (ppm)	Ion Species	Score
C28 H18 O11	TRUE	530.0834	530.0849	2.82	C28 H19 O11	93.25
C35 H14 O6		530.0834	530.079	-8.26	C35 H15 O6	71.27

--- End Of Report ---

Figure S13. HRESIMS spectrum of compound **2**

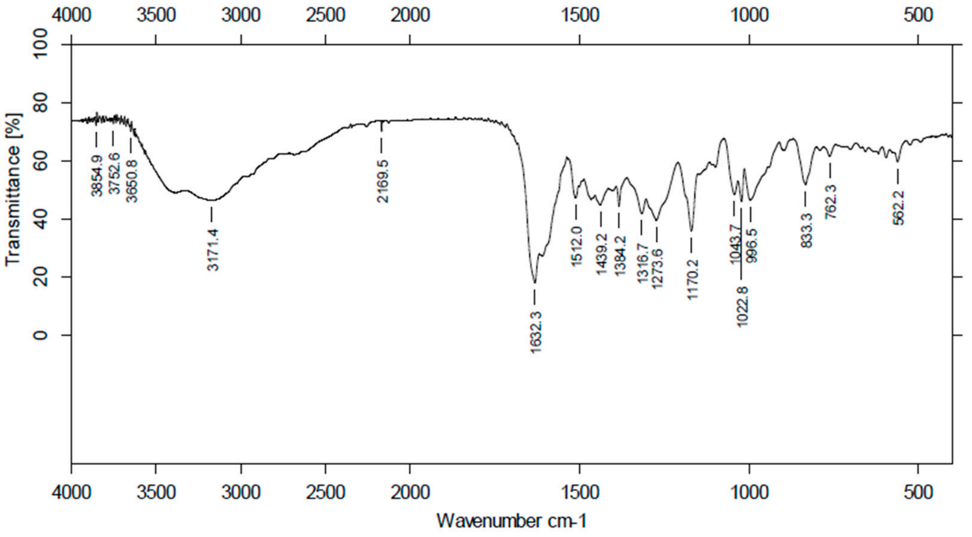

Figure S14. IR spectrum of compound **2**

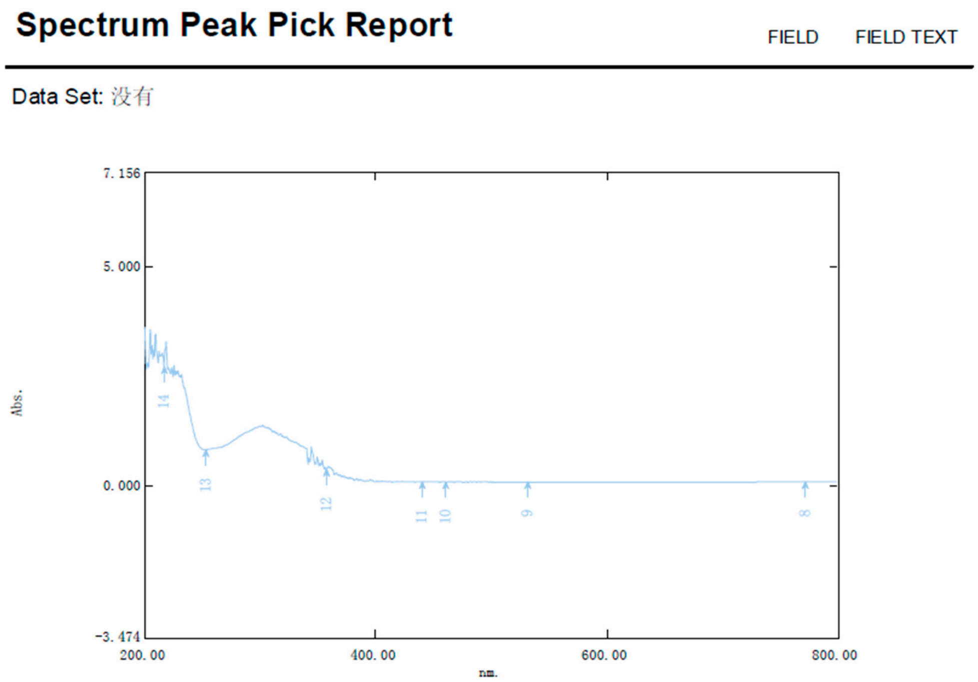

Figure S15. UV spectrum of compound **2**

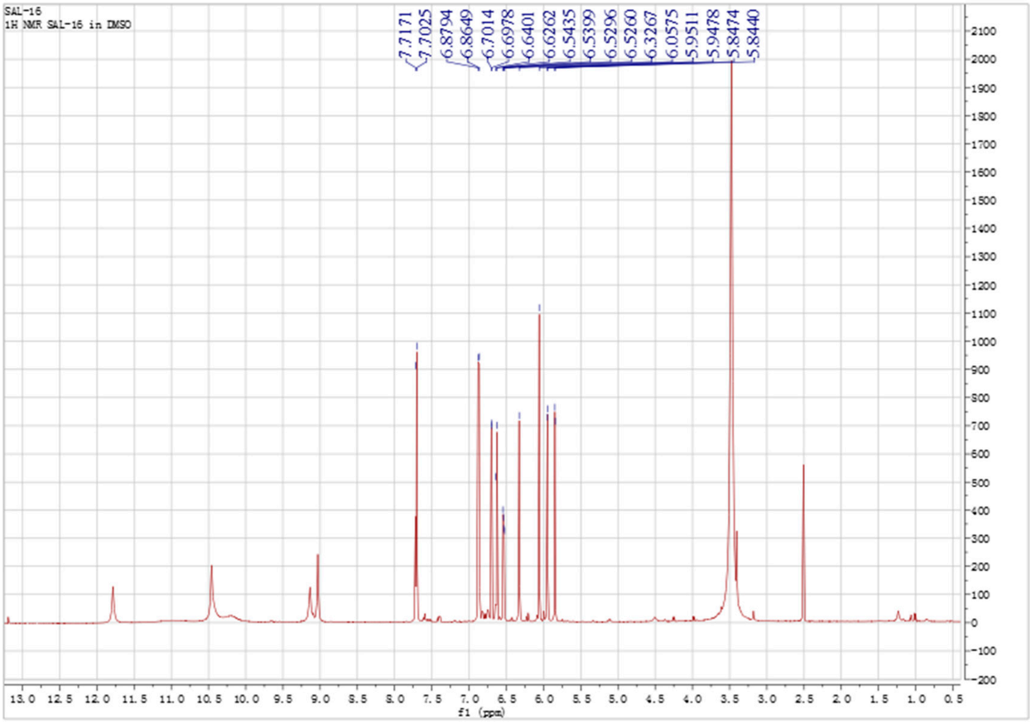

Figure S16. ^{1}H NMR spectrum (600 MHz, DMSO-d_6) of compound **3**

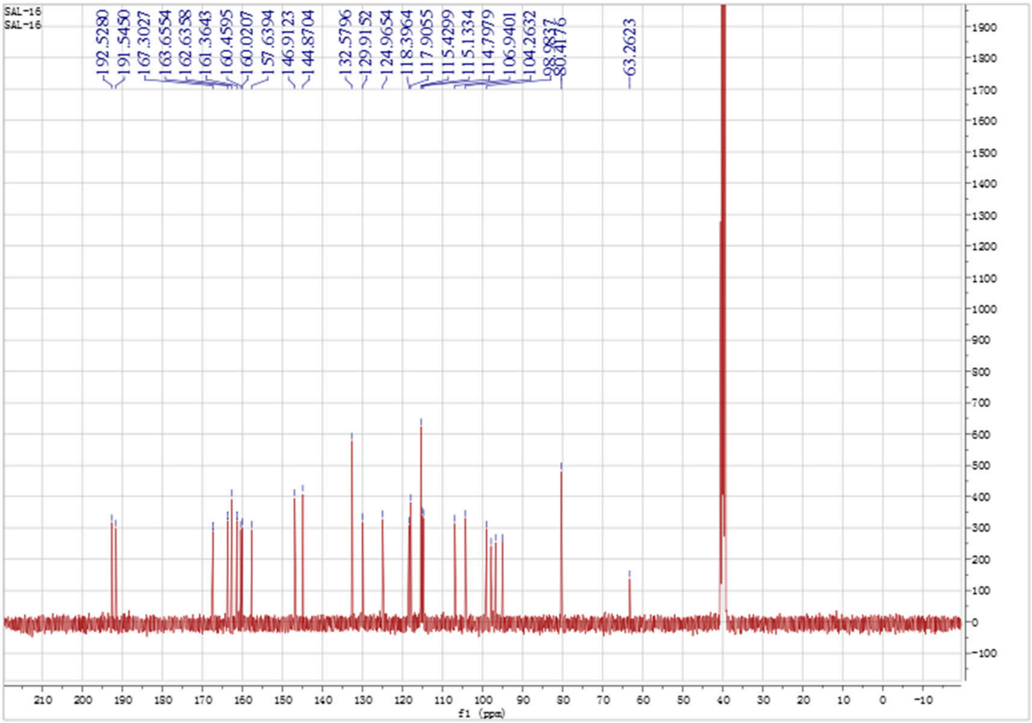

Figure S17. ^{13}C NMR spectrum (100 MHz, DMSO-d_6) of compound **3**

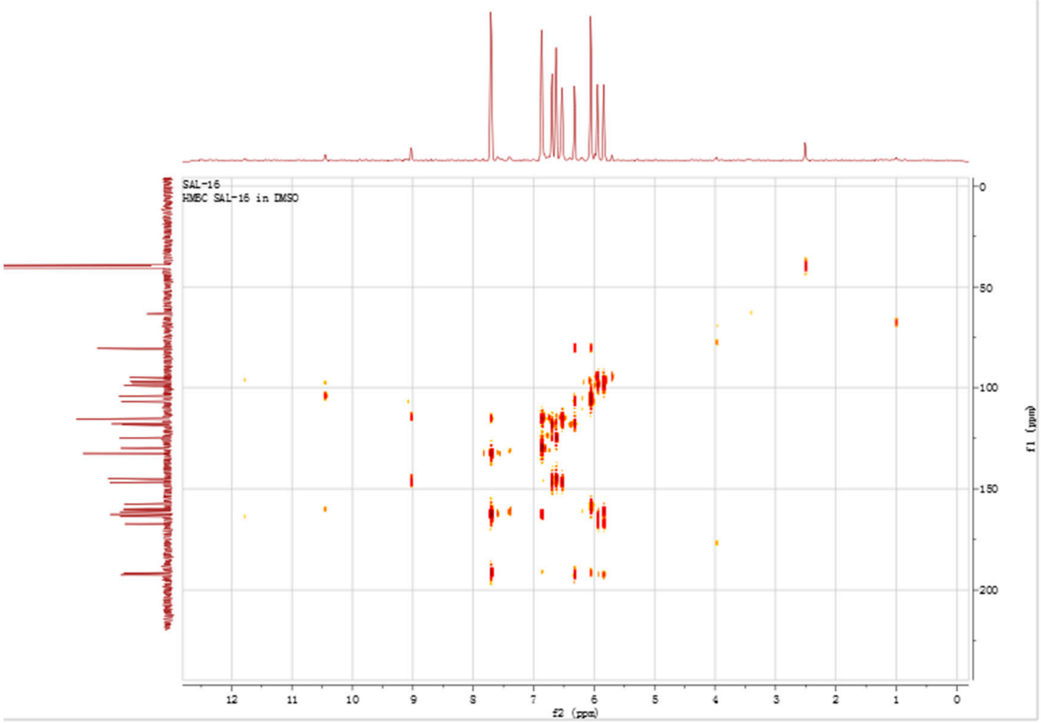

Figure S18. HMBC spectrum (600 MHz, DMSO-d_6) of compound **3**

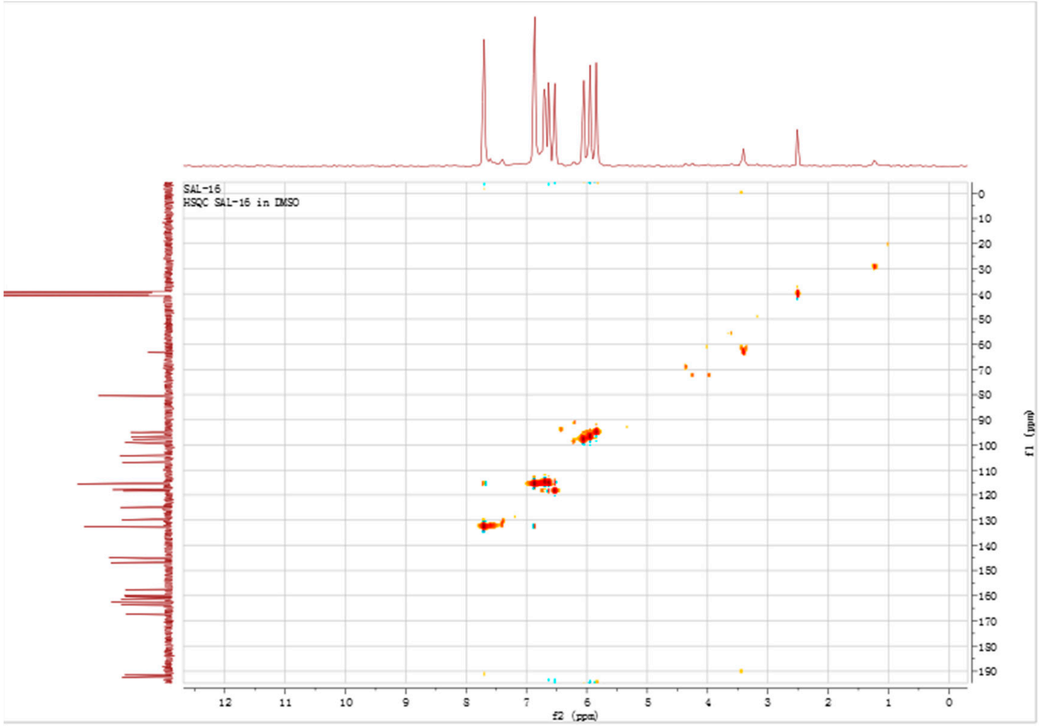

Figure S19. HSQC spectrum (600 MHz, DMSO-d_6) of compound **3**

User Spectra

Fragmentor Voltage	Collision Energy	Ionization Mode
75	0	ESI

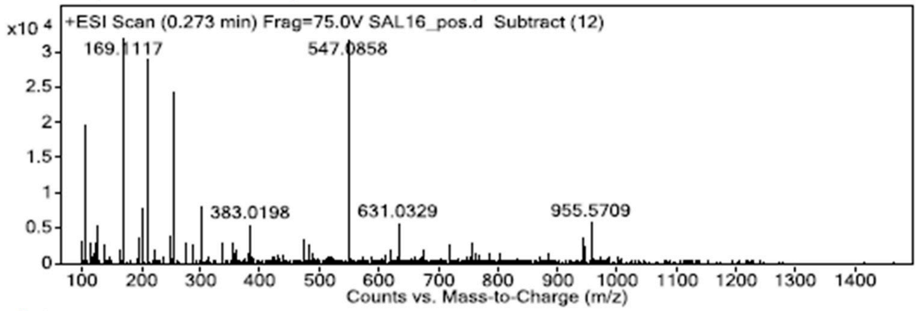

Peak List

m/z	z	Abund	Formula	Ion
103.0331		19870.1		
103.0485		8625.3		
107.041		13849.2		
107.0548		6184		
124.5455		5622.2		
169.0128		34046.2		
169.0306		12893.6		
169.1117		76825.3		
169.1306		15021.2		
201.03		7858		
210.0395		29125.1		
210.0592		12614.5		
247.025		4167.6		
253.0649		24470.5		
298.9691		8124.5		
383.0198		5587.8		
547.0858	1	31799.9	C28 H19 O12	(M+H)+
547.117	1	21631.3		
548.0873	1	10467	C28 H19 O12	(M+H)+
548.1193	1	5206.1		
631.0329		5778		
941.5485		3849.2		
955.5709		5877.1		
955.6147		5561.9		

Formula Calculator Element Limits

Element	Min	Max
C	0	60
H	0	120
O	0	15
N	0	0
S	0	0
Cl	0	0
P	0	0
B	0	0
F	0	0
Cu	0	0
Co	0	0

Formula Calculator Results

Formula	Best	Mass	Tgt Mass	Diff (ppm)	Ion Species	Score
C28 H18 O12	TRUE	546.0785	546.0798	2.44	C28 H19 O12	91.68
C35 H14 O7		546.0785	546.074	-8.32	C35 H15 O7	66.59

--- End Of Report ---

Figure S20. HRESIMS spectrum of compound **3**

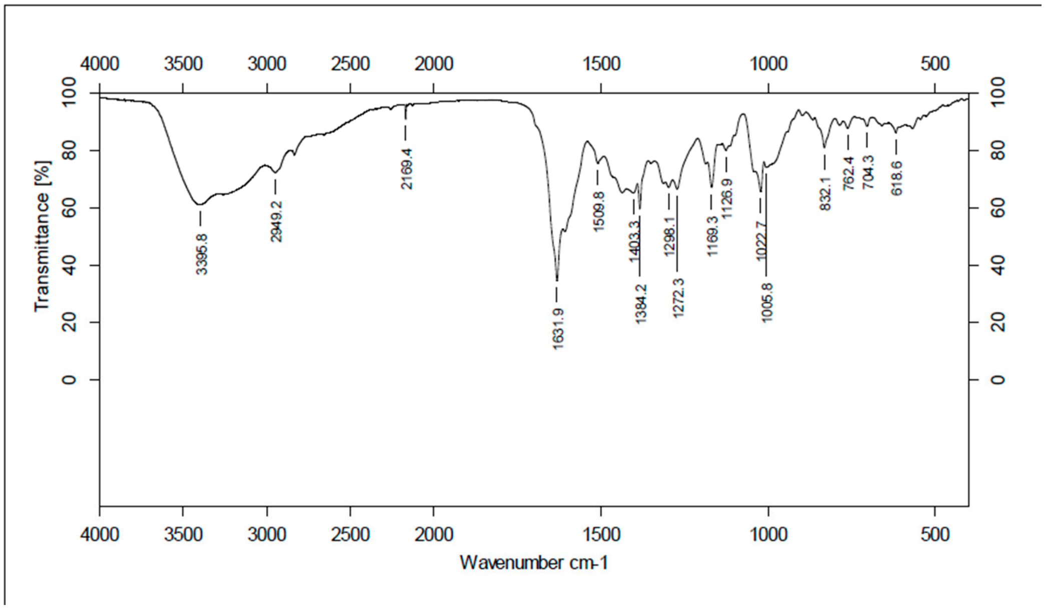

Figure S21. IR spectrum of compound **3**

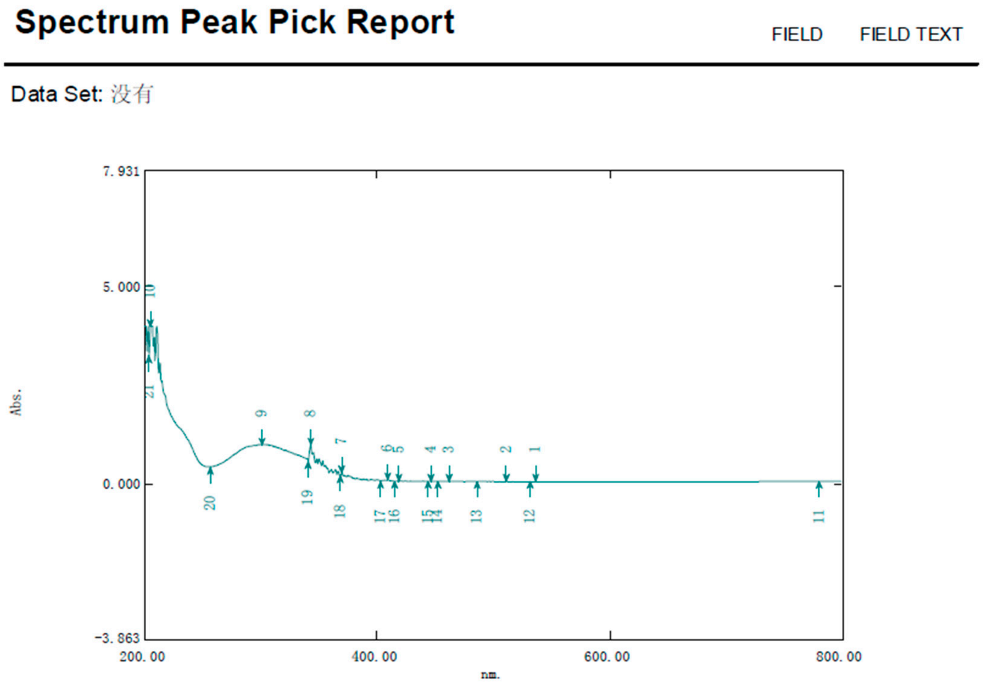

Figure S22. UV spectrum of compound **3**

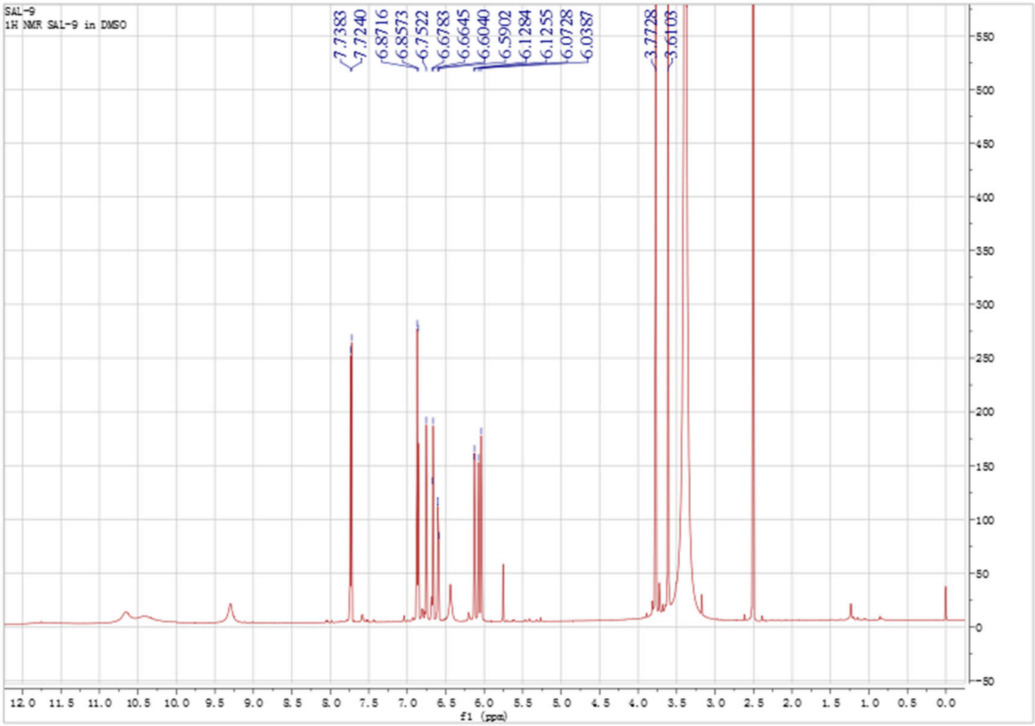

Figure S23. ^{1}H NMR spectrum (600 MHz, DMSO-d_6) of compound **4**

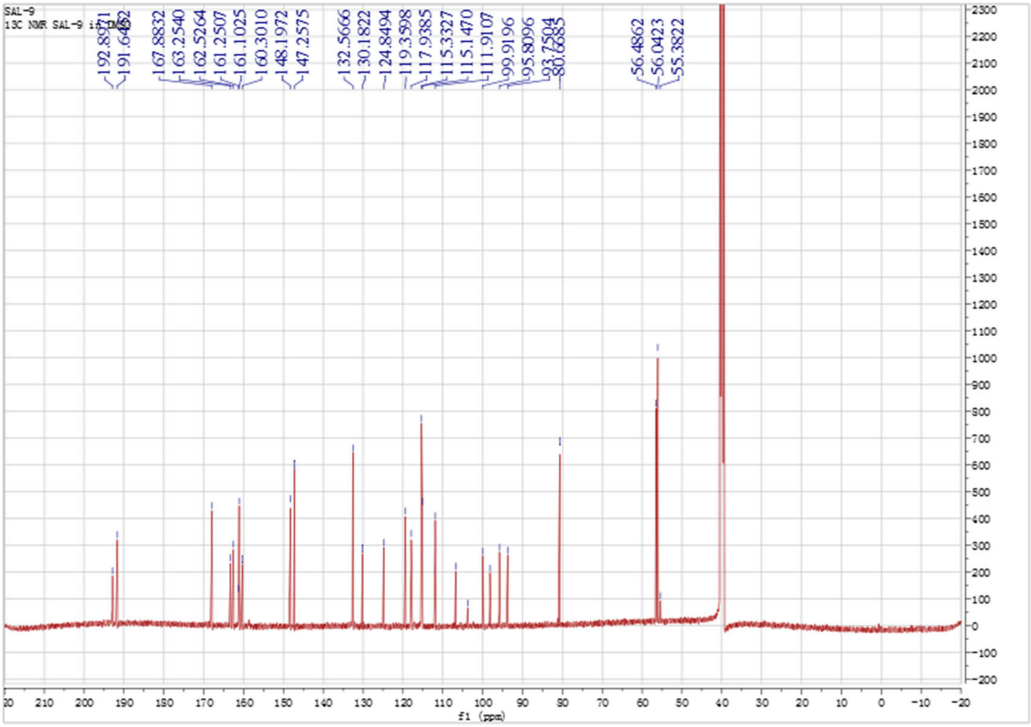

Figure S24. ^{13}C NMR spectrum (150 MHz, DMSO-d_6) of compound **4**

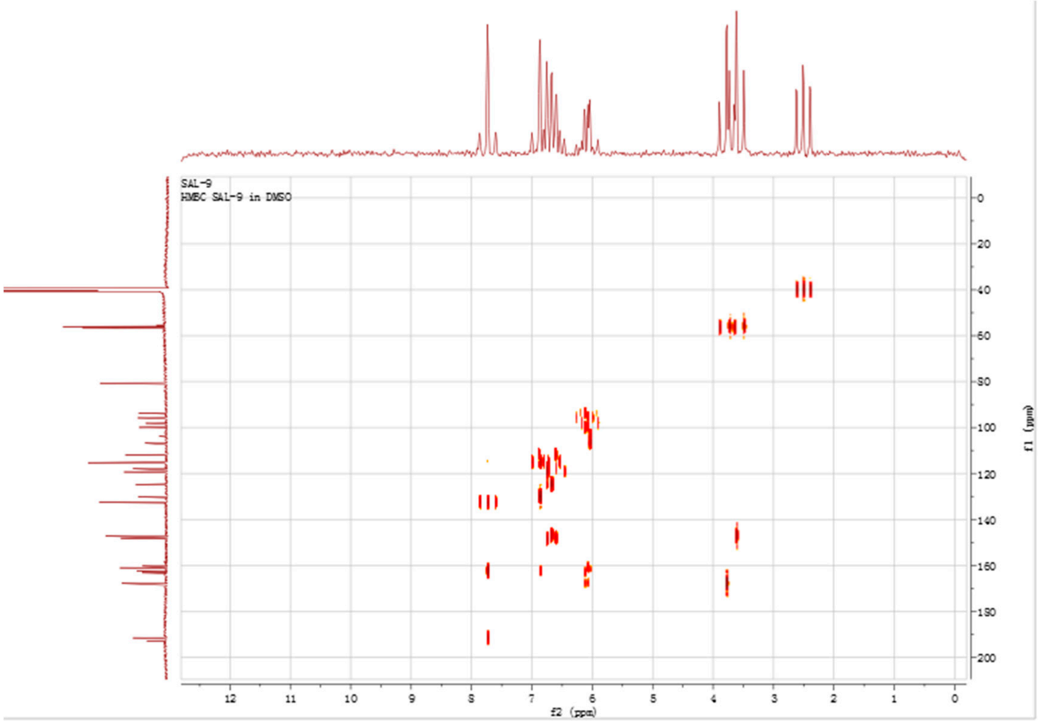

Figure S25. HMBC spectrum (600 MHz, DMSO-d_6) of compound **4**

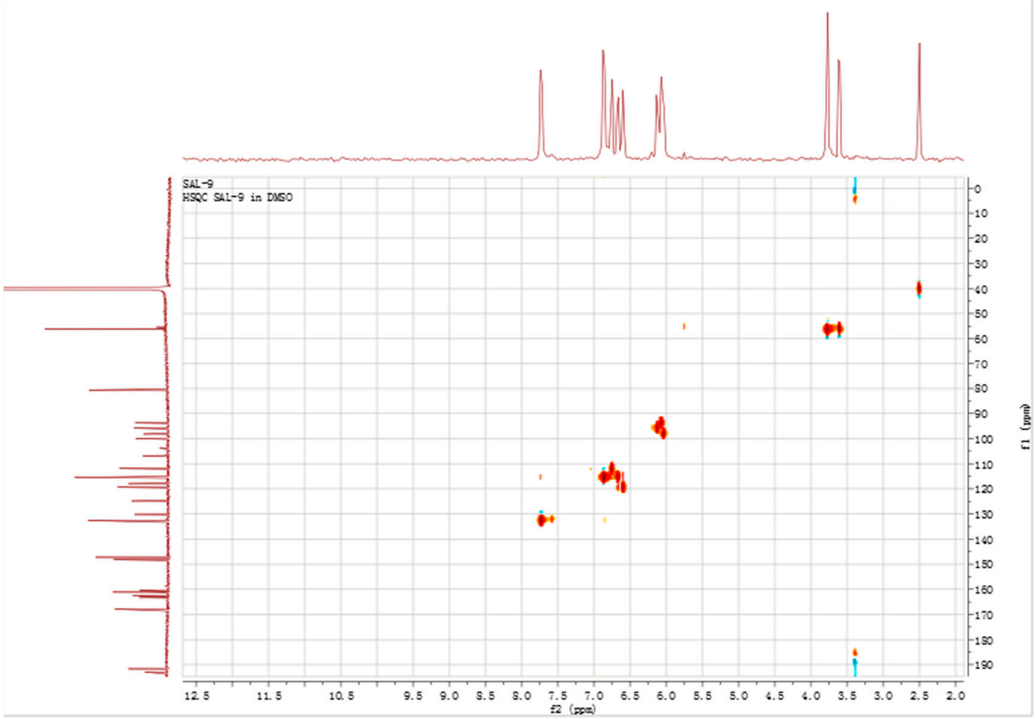

Figure S26. HSQC spectrum (600 MHz, DMSO-d_6) of compound **4**

User Spectra

Fragmentor Voltage	Collision Energy	Ionization Mode
75	0	ESI

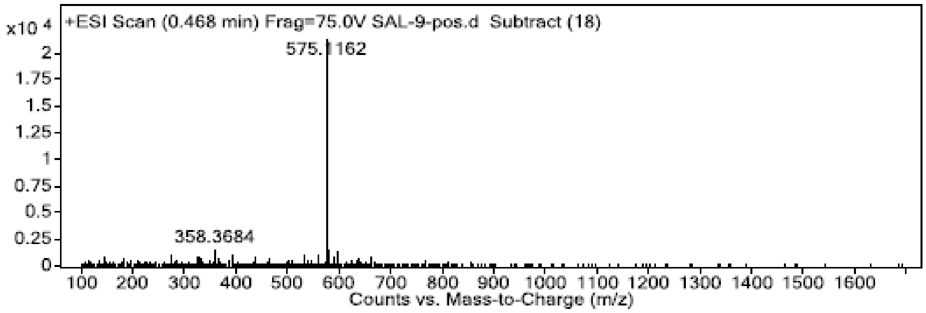

Peak List

m/z	z	Abund	Formula	Ion
273.0393		1073		
358.3684		1638		
557.1054		1090		
575.1162	1	21268.7	C30 H23 O12	(M+H)+
575.1465	1	13164.8		
575.2111		1373.6		
576.1204	1	6238.3	C30 H23 O12	(M+H)+
576.1531	1	3833.2		
577.123	1	1621.7	C30 H23 O12	(M+H)+
577.1516	1	1118.7		
597.0977		1449.6		

Formula Calculator Element Limits

Element	Min	Max
C	0	60
H	0	120
O	0	30
N	0	0
S	0	0
Cl	0	0
P	0	0
B	0	0
F	0	0
Cu	0	0
Co	0	0

Formula Calculator Results

Formula	Best	Mass	Tgt Mass	Diff (ppm)	Ion Species	Score
C30 H22 O12	TRUE	574.109	574.1111	3.77	C30 H23 O12	89.68
C37 H18 O7		574.109	574.1053	-6.46	C37 H19 O7	68.86

Qualitative Analysis Report

Formula Calculator Results

Formula	Best	Mass	Tgt Mass	Diff (ppm)	Ion Species	Score
C30 H22 O12	TRUE	574.1112	574.1111	-0.08	C30 H21 O12	98.97

--- End Of Report ---

Figure S27. HRESIMS spectrum of compound **4**

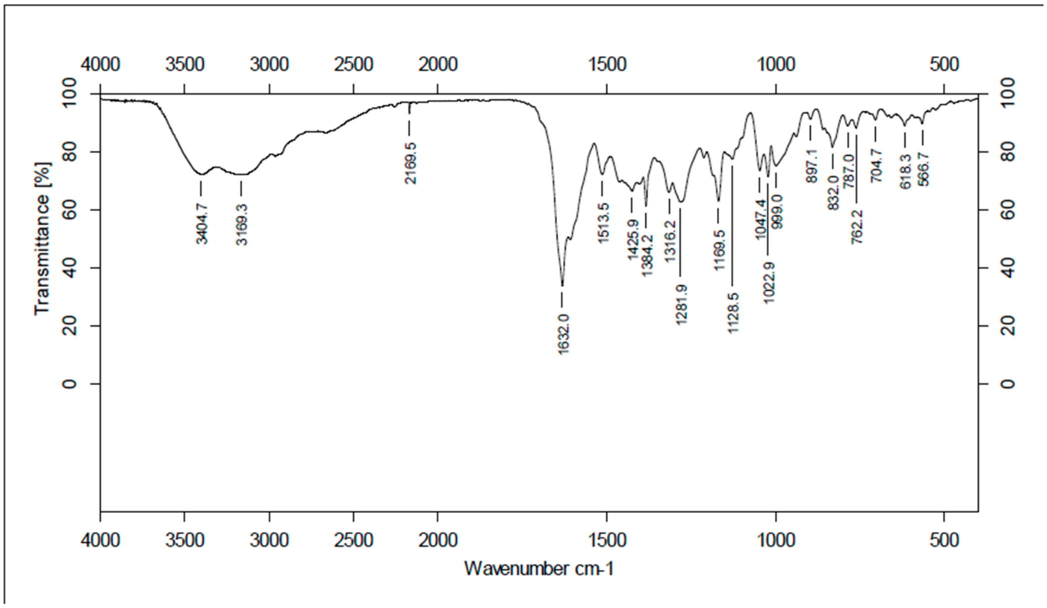

Figure S28. IR spectrum of compound **4**

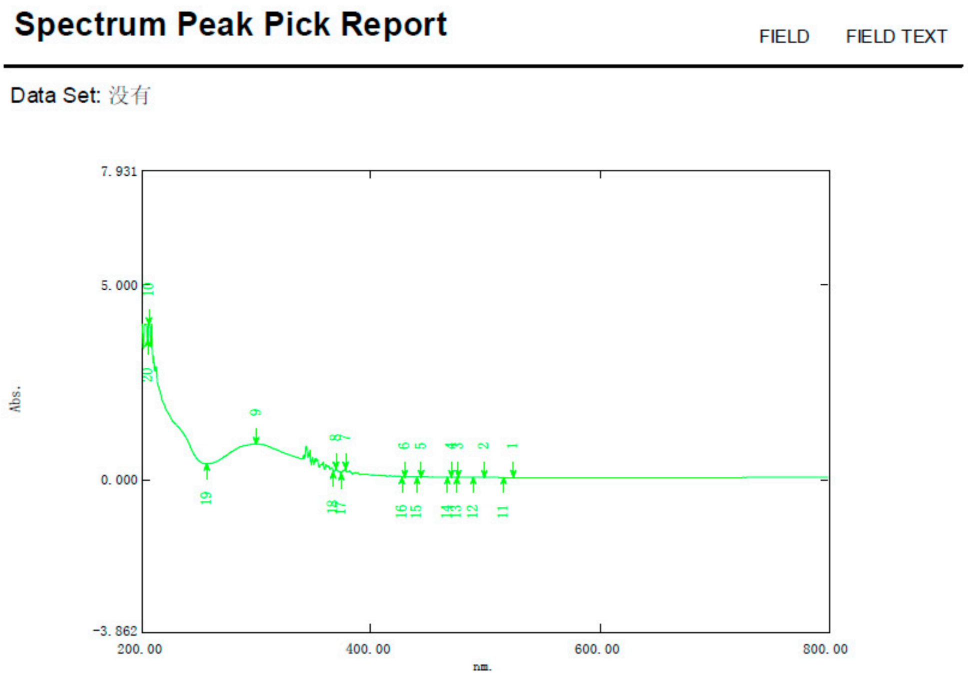

Figure S29. UV spectrum of compound **4**
